# STOP-RIO study: protocol for a randomised, controlled, open-label non-inferiority trial to test the safety of discontinuation of riociguat after balloon pulmonary angioplasty in chronic thromboembolic pulmonary hypertension

**DOI:** 10.1136/bmjopen-2026-120820

**Published:** 2026-08-07

**Authors:** Tamara Carina Rodenburg, Sanne Boerman, Marco Post, Harm-Jan Bogaard, Jurjan Aman

**Affiliations:** 1 Amsterdam Cardiovascular Sciences, Pulmonary Hypertension and Thrombosis, Amsterdam UMC, Location VUMC, Amsterdam, The Netherlands; 2 Respiratory Medicine, Sint Antonius, Nieuwegein, The Netherlands; 3 Cardiology, Saint Antonius, Nieuwegein, The Netherlands; 4 Cardiology, UMC Utrecht, Utrecht, The Netherlands

**Keywords:** Cardiovascular Disease, Hemodynamics, Thromboembolism, Pulmonary Disease

## Abstract

**Introduction:**

Balloon pulmonary angioplasty (BPA) improves haemodynamics and outcomes in patients with inoperable chronic thromboembolic pulmonary hypertension (CTEPH). Riociguat is frequently initiated before BPA to reduce procedural risks. However, evidence supporting continuation after successful BPA is lacking and current guidelines provide no criteria for treatment discontinuation. Consequently, many patients remain on long-term riociguat despite uncertain benefit, potential adverse effects and high costs.

**Methods and analysis:**

STOP-RIO is a multicentre, open-label, randomised, non-inferiority (NI) trial conducted in the two Dutch CTEPH expert centres. Seventy-four adult CTEPH patients treated with riociguat monotherapy with a mean pulmonary artery pressure (mPAP) <30 mmHg post-BPA will be randomised (1:1) to stop or continue the medication. The primary endpoint is the between-group difference in baseline-adjusted mPAP at rest measured by right heart catheterisation at 16 weeks. NI will be tested for the primary endpoint and for the two secondary endpoints highest in hierarchy N-terminal pro-B-type natriuretic peptide and 6-min walk distance. Additional secondary endpoints include haemodynamic parameters, safety outcomes, patient-reported outcomes and healthcare use. The primary analysis will be performed in a per-protocol population with secondary intention-to-treat and exploratory analyses. A prospective health economic evaluation will be conducted from a societal perspective.

**Ethics and dissemination:**

The study is conducted in accordance with the Declaration of Helsinki and Good Clinical Practice and has received ethical approval. Written informed consent will be obtained from all participants. Results will be disseminated through peer-reviewed publication and shared with patient organisations and scientific congress.

**Trial registration number:**

EU Clinical Trials Register: 2024–5 19 225-38-00.

**Strengths and limitations of this study**

- This is a randomised, non-inferiority trial addressing a clinically relevant and unresolved question in chronic thromboembolic pulmonary hypertension (CTEPH) management.
- The study uses mean pulmonary artery pressure as a guideline-aligned and clinically interpretable primary endpoint.
- The multicentre design enhances generalisability within the Western CTEPH population.
- Most existing evidence on riociguat discontinuation originates from Japanese cohorts, where haemodynamic responses to balloon pulmonary angioplasty are generally more pronounced, underscoring the importance to evaluate a discontinuation strategy in a European context.
- The open-label design may influence subjective outcomes, although the primary haemodynamic endpoint is the objective.

## Introduction

 Chronic thromboembolic pulmonary hypertension (CTEPH) is a progressive disease resulting from persistent pulmonary artery obstruction and vascular remodelling, leading to elevated pulmonary artery pressure (PAP), right heart strain and increased mortality.^1 2^ In the European Union, prevalence is estimated at 30–50 per million.^1^ While pulmonary endarterectomy (PEA) is the preferred treatment, ~40% of patients are ineligible and undergo balloon pulmonary angioplasty (BPA), often preceded by pulmonary hypertension (PH) therapy to reduce PAP, lower bleeding risks of BPA and improve right ventricular function.^1–3^

Riociguat, a soluble guanylate cyclase stimulator, is the only approved drug for inoperable or persistent/recurrent CTEPH, demonstrating efficacy in the CHEST trials.^3–6^ Despite established benefits of riociguat in CTEPH, there is limited evidence supporting its continuation after BPA. Current guidelines allow for discontinuation after successful BPA but provide no definition of ‘success’ or criteria for patient selection.^1^ Consequently, many patients remain on riociguat indefinitely, despite side effects (eg, hypotension and dizziness), high societal costs (€33 000–51 000 per patient per year) and limited evidence to support long-term use.^7 8^

**Figure 1:**
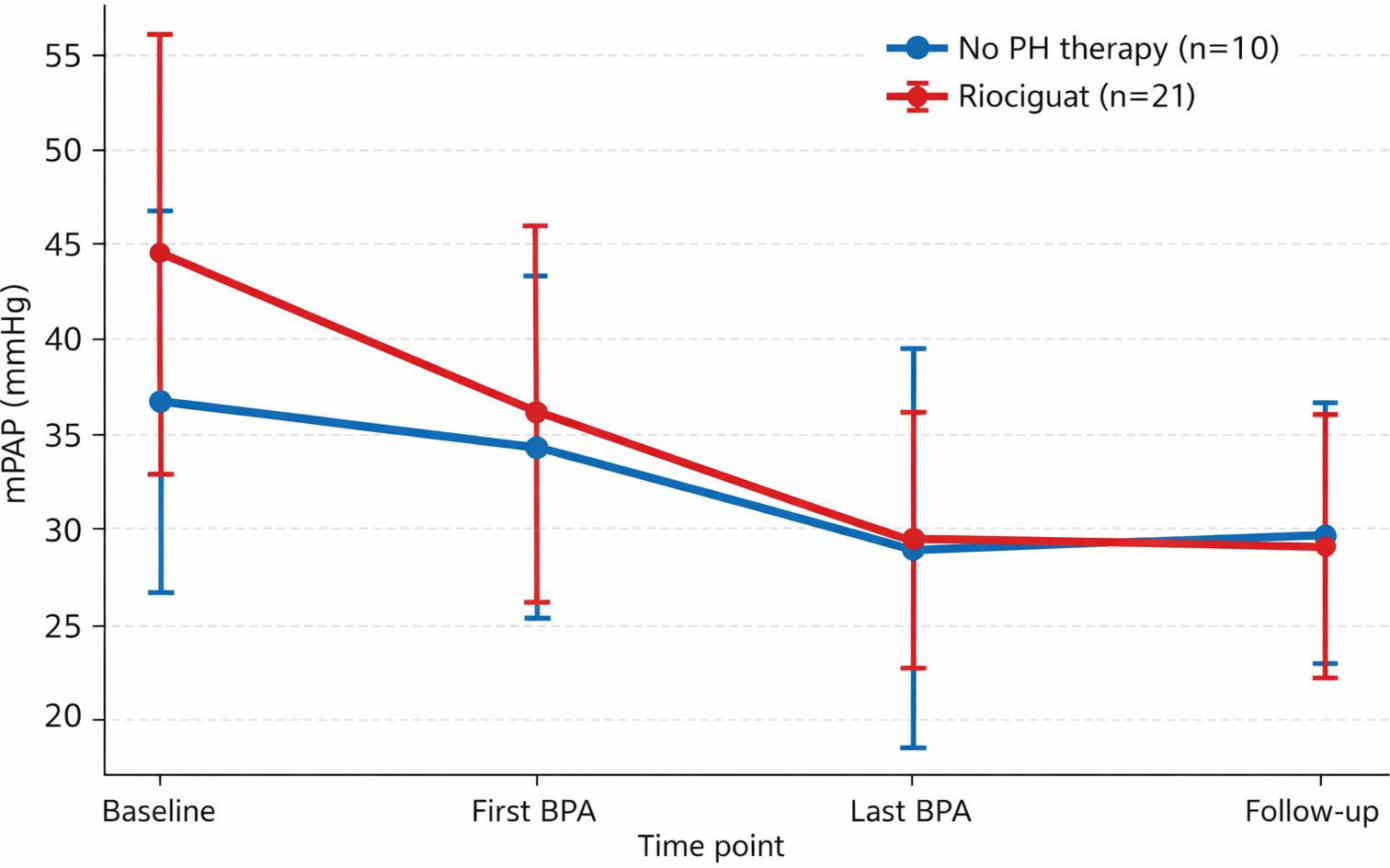
mPAP before, during and after BPA in patients treated with riociguat (red, n=21) and in patients without PH-targeted therapy (blue, n=10). In the riociguat group, baseline measurement was obtained prior to treatment initiation. Mean (±SD) mPAP values in the riociguat group were 44.4±11.7 mmHg at baseline, 35.9±10.0 mmHg at first BPA, 29.3±6.7 mmHg at last BPA and 29.0±7.0 mmHg at follow-up. Corresponding values in the no-therapy group were 36.6±10.1 mmHg, 34.2±9.0 mmHg, 28.7±10.8 mmHg and 29.6±6.9 mmHg. BPA, balloon pulmonary angioplasty; mPAP, mean pulmonary artery pressure; PH, pulmonary hypertension.

Preliminary data from Amsterdam UMC suggest that, following BPA, mean PAP (mPAP) is similar in patients treated with and without riociguat, implying that PAP reduction results primarily from desobstruction (figure 1). Another retrospective study also showed no significant difference in mPAP 1 year post-BPA between patients who discontinued PH medication and those who never received it.^9^ More recently, the multicentre, double-blind, randomised THERAPY-HYBRID-BPA trial evaluated the discontinuation of riociguat after BPA in 74 inoperable patients with CTEPH with normalised resting haemodynamics.^10^ Discontinuation did not result in a rebound increase in resting mPAP nor in differences in 6-min walk distance (6MWD) or B-type natriuretic peptide (BNP) levels compared with continuation. The primary endpoint, peak cardiac index during exercise, declined in the discontinuation group. However, translation to a Western population is limited by differences in patient characteristics and treatment strategies, while the clinical relevance of a higher peak exercise cardiac index remains uncertain, as riociguat may affect cardiac index independently from its effects on the pulmonary circulation, through direct cardiac effects or systemic vasodilation.^11–13^

Given the absence of prospective evidence in Western populations and the lack of standardised discontinuation criteria, this randomised non-inferiority (NI) trial aims to assess the safety and feasibility of stopping riociguat after BPA in patients with mPAP <30 mmHg (primary objective), including a prospective health economic evaluation of the societal and patient-related benefits of riociguat discontinuation (secondary objective).

## Methods

### Study design

This is a multicentre, open-label, randomised, controlled NI trial will include 74 CTEPH patients who reached an mPAP <30 mmHg post-BPA, comparing discontinuation versus continuation of riociguat post-BPA. The trial will be conducted at Amsterdam UMC and St. Antonius Hospital, the two Dutch national expert centres for CTEPH. To enhance generalisability, we aim to include additional European centres. Diagnosis of CTEPH, indication for BPA and initiation of riociguat therapy are a part of routine clinical care and fall outside the scope of the trial intervention. The estimated duration of the study is 2.5 years.

Ethical approval was obtained from the Medical Ethics Review Committee Amsterdam UMC (2025.0927) for the trial registered in the Clinical Trials Information System under registration number 2024–5 19 225-38-00.

### Study population

The study population consists of adult patients with CTEPH who are treated with riociguat monotherapy and have completed their final BPA session 6 months prior to screening. Patients are recruited from routine follow-up visits at the participating centres.

Eligible patients must have demonstrated an mPAP <30 mmHg on right heart catheterisation (RHC) performed within 3 months prior to inclusion. If no recent RHC is available, it will be performed after informed consent. Patients with an mPAP ≥30 mmHg are excluded.

Inclusion criteria include: age ≥18 years; diagnosis of CTEPH according to prevailing European guidelines; treatment with riociguat monotherapy initiated prior to or within 2 weeks after the first BPA session; availability of a documented post-BPA follow-up RHC performed within the last 3 months or an anticipated post-BPA follow-up RHC with an mPAP <30 mmHg as determined by the treating physician’s judgement; ability to comply with study procedures; and provision of written informed consent.

Exclusion criteria include: WHO functional class IV; cardiac index <2.2 L/min/m²; prior PEA; post-BPA follow-up RHC within 3 months indicating an mPAP ≥30 mmHg; participation in another interventional drug trial; severe comorbidities limiting life expectancy to less than 1 year; or any condition that, in the investigator’s judgement, precludes safe participation.

### Randomisation and allocation

After confirmation of eligibility and completion of baseline assessments, participants are randomised in a 1:1 ratio to either discontinuation or continuation of riociguat. Randomisation is performed in CastorEDC (Ciwit B.V., Amsterdam, The Netherlands) using block randomisation with variable block sizes. Stratification is applied by riociguat dose at inclusion (daily dose of <6 mg vs ≥6 mg) and time since last BPA, distinguishing incident patients (scheduled for RHC as part of standard clinical follow-up) from prevalent patients (those in long-term follow-up, screened by transthoracic echocardiography (TTE); last RHC performed more than 3 months ago). Due to the nature of the intervention, the trial is conducted as an open-label study. Blinding was considered impractical given the recognisable side effects of riociguat and the objective nature of the majority of primary and key secondary outcomes. However, all physicians performing outcome measurements will be blinded to treatment allocation.

### Interventions

Participants allocated to the control group continue riociguat at the pre-inclusion dose, administered three times a day in accordance with current clinical guidelines.^5 6^ Dose adjustments for hypotension or adverse effects are permitted at the discretion of the treating physician. Participants allocated to the intervention group discontinue riociguat following a structured tapering schedule over 6 days. Treatment is reduced from 2.5 mg three times a day to 2.0 mg three times a day for 2 days, followed by 1.0 mg three times a day for 2 days, after which the medication is discontinued. Participants receiving a lower dose at baseline will enter the tapering schedule at the corresponding step and may skip preceding dose-reduction steps. In case of clinical worsening during follow-up, riociguat is re-initiated at 1.0 mg three times a day for 2 weeks, followed by up-titration to the pre-inclusion dose as tolerated, in accordance with clinical guidelines.^5 6^

### Follow-up and safety monitoring

**Figure 2:**
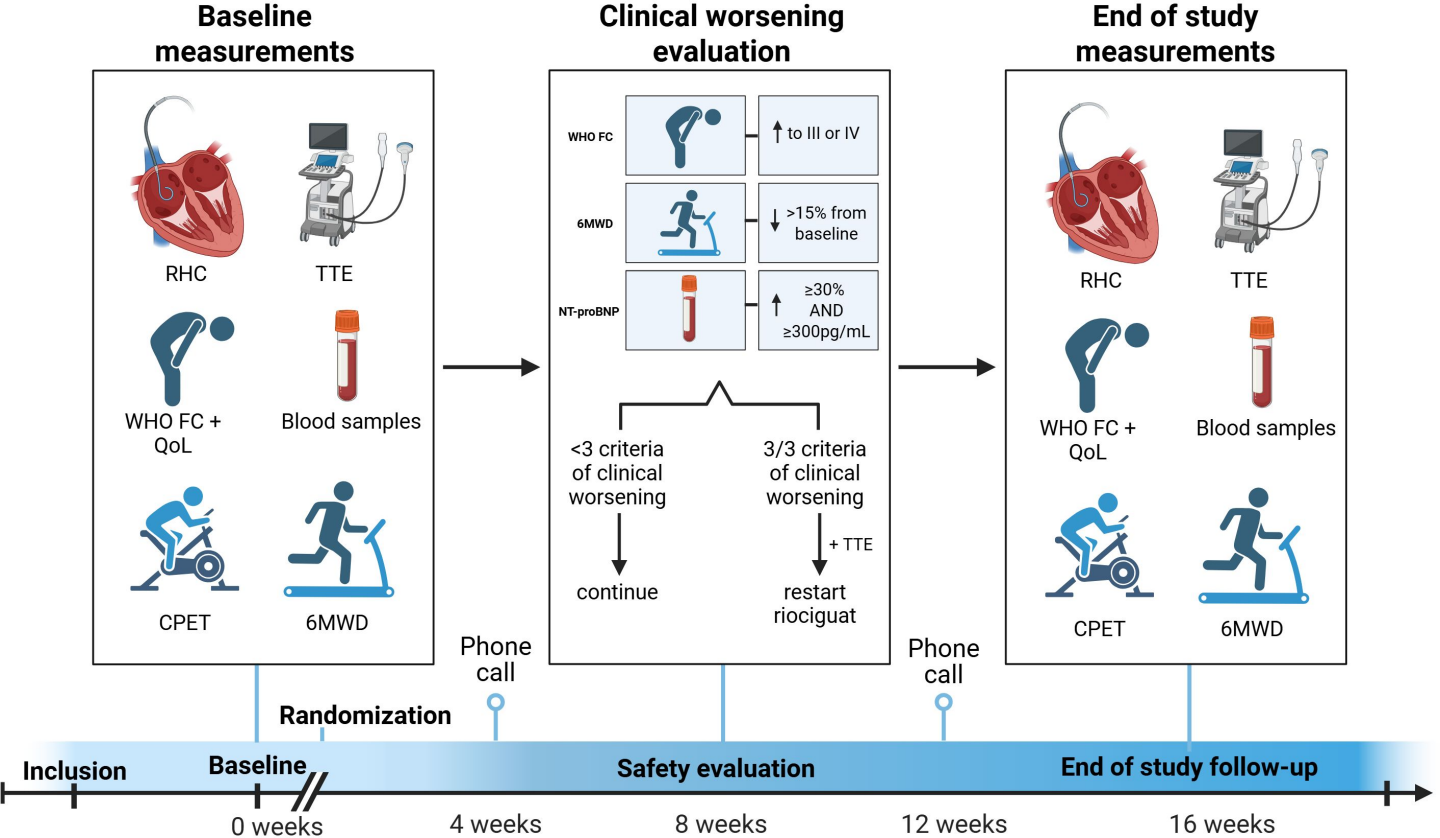
Outline of study protocol. At the end of study follow-up, participants will also complete a survey assessing satisfaction with the discontinuation strategy. 6MWD, 6-min walking distance; CPET, cardiopulmonary exercise testing; FC, functional class; QoL, quality of life; RHC, right heart catheterisation; TTE, transthoracic echocardiogram.

The study protocol is outlined in figure 2. Baseline assessments include RHC, cardiopulmonary exercise testing, 6MWD with Borg dyspnoea score, TTE, laboratory testing (including N-terminal pro-BNP (NT-proBNP)), and quality of life questionnaires. These assessments will be repeated at 16 weeks (end of study). Monthly follow-up is performed by outpatient visits or telephone contact to assess symptoms, medication adherence and adverse events (AEs). AEs are collected using open-ended and structured queries, including predefined symptoms such as dizziness and diarrhoea. Blood pressure is measured at each contact; hypotension (≤90/60 mmHg) is graded (grade 1–5) according to Common Terminology Criteria for Adverse Events (CTCAE) v5.0.^14^ Participants have 24/7 access to their PH care team.

A predefined safety evaluation is performed at week 8 (±3 weeks). Clinical worsening is defined as the simultaneous occurrence of all three of the following criteria: (1) >15% decrease in 6MWD, (2) progression to WHO functional class III/IV, and (3) increase in NT-proBNP of ≥30% to a level of ≥300 pg/mL. This definition is adapted from prior pulmonary arterial hypertension (PAH) and CTEPH trials and aligns with criteria used in the STELLAR trial.^3 15–18^ If clinical worsening is confirmed, TTE is performed and riociguat is reinitiated in the discontinuation group. Unscheduled evaluations may be performed at the investigator’s discretion in case of suspected clinical deterioration.

At study completion, participants complete a survey assessing satisfaction with the discontinuation strategy. Riociguat may be reintroduced after the end-of-study RHC at the discretion of the treating physician and patient.

### Objectives and endpoints

The primary endpoint is the between-group difference in mPAP at rest, measured by RHC at 16 weeks. Secondary endpoints for the primary study objective are outlined in table 1.

**Table 1:** Secondary outcomes for the primary study objective

Objectives	Secondary endpoints
To investigate if discontinuation of riociguat is non-inferior to its continuation in CTEPH patients with mPAP <30 mmHg post-BPA	Efficacy endpoints: Between-group differences at 16 weeks for the following parameters in hierarchical order: NT-proBNP (assessed for non-inferiority) 6MWD (assessed for non-inferiority) PVR Cardiac index Between-group differences at 16 weeks in exercise capacity in post hoc analysis: CPET variables: oxygen uptake, workload, heart rate, respiratory exchange ratio, ventilation, oxygen saturation, oxygen pulse, equivalent carbon dioxide Change in modified Borg dyspnoea scale Safety endpoints: Between-group differences at 16 weeks in the following parameters: Number of adverse events and severe adverse events Proportion of participants with mPAP ≥38 mmHg at RHC Proportion of participants meeting the clinical worsening criteria at week 8 Feasibility of the strategy: Is TTE sufficient to replace RHC at the end of study follow-up? The correlation between TTE and RHC parameters Right ventricular systolic pressure Tricuspid annular plane systolic excursion Proportion of participants missed by TTE with mPAP ≥38 mmHg at 16 weeks RHC Is the discontinuation strategy feasible for implementation in clinical practice? Proportion of participants missed by the clinical worsening criteria with mPAP ≥38 mmHg at 16 weeks Participants' satisfaction with the protocol

BPA, balloon angioplasty; CPET, cardiopulmonary exercise testing; CTEPH, chronic thrombo-embolic pulmonary hypertension; mPAP, mean pulmonary artery pressure; 6MWD, 6-min walking distance; NT-proBNP, N-terminal pro-B-type natriuretic peptide; PVR, pulmonary vascular resistance; RHC, right heart catheterisation; TTE, transthoracic echocardiography; WHO, world health organisation.

### Non-inferiority margin

The NI margin for mPAP was set at 4 mmHg. This margin reflects a clinically relevant rebound increase while remaining below established treatment thresholds. In our target population, the mean mPAP was approximately 23.6 mmHg. An NI margin of 4 mmHg ensures that the upper limit of the 95% CI remains below 30 mmHg, the threshold for initiating PH therapy (online supplemental figure).^1^

To place this margin in the context of expected interindividual variability, the distribution of mPAP at follow-up was estimated using a pooled SD derived from longitudinal haemodynamic data. Based on this variability, 97.5% of patients are expected to have an mPAP below 38 mmHg at follow-up (23.6+4 + 2×4.9), a threshold that has been associated with worse long-term outcomes in CTEPH.^19^ This approach aims to preserve haemodynamic safety at both the group and individual patient level (online supplemental figure).

The SD of mPAP was estimated from preliminary cohort data in patients meeting the inclusion criterion of mPAP <30 mmHg at 6 months post-BPA (SD 3.43 mmHg). To account for longitudinal variability beyond 6 months, unpublished follow-up data from BPA-treated and PEA-treated patients followed from 6 to 18 months post-intervention, were incorporated (n=40), yielding an SD of mPAP change of 3.8 mmHg, with a correlation of −0.08. The pooled SD was calculated as: $\sqrt{\sigma^{2}combi}=\;\sqrt{3.43^{2}+\;3.8^{2}+\;\left(2\times -0.08\times \;3.43\times \;3.8\right)}=4.9$, which was used for both the sample size calculation and estimation of interindividual variability.

### Sample size calculation

The sample size calculation was performed for the primary endpoint, mPAP at 16 weeks, and for the two secondary endpoints highest in the hierarchy (NT-proBNP and 6MWD). Sample size calculations were performed using the formula: n=f(α,β)×2×σ²/d², where f(α,β) = (Φ⁻¹(α) + Φ⁻¹(β))². For the primary endpoint (mPAP at 16 weeks), assuming a one-sided α of 0.025, power of 90%, SD of 4.9 mmHg and an NI margin of 4 mmHg, 64 participants (32 per group) are required. Allowing for a 10% dropout rate, the target sample size for the primary endpoint is 70 participants.

For NT-proBNP, a NI margin of 30% was chosen, reflecting a clinically meaningful change commonly used in PH and heart failure trials.^20–24^ NT-proBNP values from preliminary data were log-transformed (mean 5.04, SD 1.15), corresponding to a NI margin of 1.51 on the log scale. This yielded a required sample size of 26 participants (13 per group) with 90% power and one-sided α=0.025; accounting for dropout, 29 participants are required. For 6MWD, a NI margin of 15% was used, consistent with widely used definitions of clinical worsening in PAH and CTEPH trials. Using a mean of 467 m and an SD of 95 m of riociguat-treated post-BPA patients derived from a study by Wiedenroth *et al*,^25^ 66 participants (33 per group) are required with 85% power and one-sided α=0.025. After allowing for 10% dropout, 74 participants are required. As the largest sample size was required for 6MWD, the total sample size was set at 74 participants.

NT-proBNP was prioritised in the hierarchical testing strategy because it is an objective biomarker that reflects haemodynamic status. Also, the planned sample size provides greater statistical power for NT-proBNP than for 6MWD.

### Statistical analysis

All analyses will be performed using R (R Foundation for Statistical Computing, Vienna, Austria). Continuous variables will be summarised as mean±SD or median (IQR) as appropriate; categorical variables as counts and percentages. Normality will be assessed using histograms and Q–Q plots. Skewed variables will be log-transformed when necessary.

The null hypothesis is that riociguat discontinuation is inferior to continuation. NI will be concluded if the upper bound of the two-sided 95% CI for the baseline-adjusted between-group difference does not exceed the predefined NI margin. A one-sided significance level of α=0.025 will be used.

A secondary analysis of the primary endpoint (mPAP at 16 weeks) will include an analysis of covariance (ANCOVA), with baseline mPAP as a covariate. Treatment group will be included as the main effect, with riociguat dose and incident/prevalent status included as fixed factors to account for stratification at randomisation. To control the type I error rate, hierarchical testing will be applied. Secondary continuous outcomes will be analysed using ANCOVA or linear regression adjusted for baseline values. Binary outcomes will be analysed using χ^2^ or Fisher’s exact tests. Ordinal outcomes will be analysed using stratified Wilcoxon tests or linear mixed models for repeated measures, as appropriate. All estimates will be reported with two-sided 95% CIs.

### Analysis set

The primary analysis will be conducted in the per-protocol population, as an intention-to-treat (ITT) analysis can be considered anticonservative in a NI study testing discontinuation. Participants who restart riociguat due to clinical worsening will be analysed in the continuation group. As a secondary analysis, we will perform an ITT analysis according to randomised allocation, reflecting the clinical discontinuation strategy including re-initiation and testing the safety of the stopping strategy. In addition, two exploratory analyses will be performed: a modified ITT analysis excluding participants who restarted riociguat from the discontinuation group to explore outcomes among patients who tolerate treatment withdrawal without re-initiation, and a subgroup analysis comparing participants who restarted riociguat with the continuation group to assess haemodynamic response following re-initiation. Non-compliant patients who meet protocol violations will be excluded as part of the modified per-protocol (PP) analysis. Missing data will not be imputed. However, participants who withdraw before the week 8 clinical worsening assessment because of clinical deterioration, or for whom the reason for withdrawal is unknown, will be conservatively classified as having experienced clinical worsening.

To account for potential loss of participants from the primary per-protocol analysis due to clinical worsening, predefined scenarios were incorporated into the statistical analysis planning. Based on power simulations, the study is expected to retain approximately 80% power if up to 35% of participants are lost from the intervention group. To mitigate the risk of insufficient power, the randomisation ratio will be reassessed after inclusion of 50% of the planned sample size. If ≥30% or ≥40% of participants in the discontinuation group have restarted riociguat at that point, the randomisation ratio will be adaptively adjusted from 1:1 to 2:1 or 3:1, respectively, in favour of the discontinuation group. If ≥50% of participants in the discontinuation group experience clinical worsening, the study will be terminated for safety reasons, as such a rate is considered clinically unacceptable.

### Health economic evaluation

A prospective economic evaluation will be conducted after completion of the trial. Costs related to medication use, healthcare consumption (including outpatient visits, hospital admissions and emergency department visits) and patient travel will be collected prospectively. Health-related quality of life will be assessed using the EuroQol five-dimensional five-level questionnaire (EQ-5D-5L) and Living with Pulmonary Hypertension Questionnaire (LPHQ). These outcomes will be incorporated into a formal health-economic evaluation to estimate cost-effectiveness in terms of quality-adjusted life years. Secondary endpoints for the secondary study objectives are outlined in table 2.

**Table 2:** Secondary endpoints for the secondary objectives

Secondary objectives	Endpoints for secondary objectives
To assess the societal impact benefits associated with riociguat discontinuation in CTEPH patients with mPAP <30 mmHg post-BPA	Economic outcomes Between-group difference in costs related to PH medication Between-group difference in healthcare consumption Frequency of visits to expert centres versus local hospitals Travel distance for medical care Number of emergency department visits Patient-related outcomes Between-group difference at 16 weeks in the following parameters: WHO functional class Quality of life questionnaire scores LPHQ EQ-5D-5L Number of predefined graded adverse events: Mean gradation of hypotension Mean gradation of dizziness Mean gradation of diarrhoea

BPA, balloon angioplasty; CTEPH, chronic thrombo-embolic pulmonary hypertension; EQ-5D-5L, EuroQol five-dimensional five-level questionnaire ; LPHQ, Living with PH questionnaire; mPAP, mean pulmonary artery pressure; PH, pulmonary hypertension.

### Data management, monitoring and confidentiality

All study data will be collected and managed using CastorEDC, a secure, web-based electronic data capture system compliant with Good Clinical Practice (GCP) and the General Data Protection Regulation. Data will be pseudonymised. Access to the study database will be restricted to authorised study personnel. Data quality is ensured through built-in validation checks, predefined range limits and regular monitoring of entered data. Source data verification will be performed for key study variables. Study data will be stored for a minimum of 15 years in accordance with local regulations.

Monitoring will be conducted by an independent team of monitors. A data safety monitoring board will not be established for this study, as it is not considered a high-risk trial and no formal interim analysis is required. The study may be subject to audit by the sponsor, ethics committees or regulatory authorities to assess compliance with GCP.

### Patient and public involvement

Patients have been involved throughout the project. The study proposal was reviewed by the Dutch Pulmonary Hypertension Association and the Pulmonary Hypertension Patient Advisory Board of Amsterdam UMC, who provided input to optimise the study design and minimise patient burden. Patient involvement is organised across three phases: study design, study conduct with feedback from participating patients, and implementation of study results with patient organisations. Participants will be surveyed about their experience at the end of follow-up, and patient organisations will be involved in dissemination and implementation of the study findings.

## Discussion

The STOP-RIO trial addresses a clinically relevant issue in management of CTEPH; whether riociguat can be discontinued after BPA in patients with favourable haemodynamics. Despite guideline statements allowing for de-escalation of PH therapy after BPA, the lack of Western prospective data has resulted in heterogeneous clinical practice and long-term continuation of riociguat.^2 8^ STOP-RIO is designed to provide evidence guiding a standardised and clinically meaningful discontinuation strategy.

A central design choice is the use of mPAP as the primary endpoint with an eligibility threshold of <30 mmHg, consistent with the threshold ≥30 mmHg associated with clinical deterioration and initiation of PH therapy after PEA,^19^ and with current consensus statements and European guideline-based haemodynamic targets after BPA.^1 26 27^ Compared with pulmonary vascular resistance (PVR) or cardiac index, mPAP offers greater clinical interpretability and is less susceptible to confounding by systemic effects of riociguat. While PVR is closely related to disease pathophysiology, its interpretation after riociguat discontinuation is confounded by the drug’s effect on the heart and systemic vascular resistance. In contrast, mPAP demonstrated a clear threshold effect between 25 mmHg and 30 mmHg in a prior study, whereas no comparable pattern was observed for PVR or cardiac index.^19^ Exclusion of patients with cardiac index <2.2 L/min/m² further limits low CO confounding. This approach differs from the THERAPY-HYBRID-BPA trial, in which exercise-derived cardiac index might be more susceptible to confounding, particularly in the context of a NI design.^10^

The NI margin was primarily chosen to target an upper limit of the 95% CI of the mean mPAP below 30 mmHg after riociguat discontinuation, consistent with current treatment targets after BPA. The 38 mmHg threshold was used to contextualise the expected interindividual variability, based on the best available prognostic evidence from patients after PEA. Although this threshold has not been validated in patients after BPA, it currently represents the most appropriate prognostic reference available. To further evaluate the clinical relevance of haemodynamic thresholds after BPA, we aim for an exploratory subgroup analysis to compare patients with an mPAP <25 mmHg and ≥25 mmHg after BPA.

Beyond individual patient outcomes, STOP-RIO addresses other organisational and economic considerations. Riociguat therapy entails high drug costs, centralised follow-up and AEs particularly harming in an older patient population with increased risk of falls and bleeding that may contribute to more healthcare use and patient burden. Prospective assessment of these parameters will allow evaluation of the broader clinical and economic impact of a riociguat discontinuation.

Although the randomised NI design, the multicentre setting within national CTEPH expert centres and the use of a guideline-aligned primary endpoint allow for a clinically relevant evaluation of riociguat discontinuation after BPA, several limitations should be acknowledged. Most existing discontinuation data originate from Japanese cohorts. Differences in patient characteristics, treatment strategies and haemodynamic responses to BPA may limit the generalisability of these findings to Western populations,^28^ underscoring the importance of evaluating this strategy in a European setting. We therefore aim to include additional European centres. The open-label design may influence subjective outcomes, although it is unlikely to affect the primary endpoint. In addition, no independent blinded endpoint adjudication committee was included; however, clinical worsening and riociguat re-initiation are based on predefined protocol criteria. Finally, the 16-week follow-up limits assessment of the long-term clinical and health economic consequences of riociguat discontinuation. Therefore, we aim for a long-term follow-up study, including an extended health economic evaluation.

Overall, STOP-RIO aims to fill an important evidence gap in post-BPA management and optimise long-term care for CTEPH patients.

## Ethics, dissemination and data availability

The study is conducted in accordance with the Declaration of Helsinki and GCP guidelines. Ethical approval was obtained from the Medical Ethics Review Committee Amsterdam UMC (2025.0927) and written informed consent will be obtained from all participants prior to any study procedures. Any substantial protocol amendments will be submitted for ethics approval prior to implementation.

Study results will be disseminated through publication in peer-reviewed journals and presentation at national and international scientific meetings. Findings will also be shared with participating patients and relevant patient organisations in an accessible format. Individual participant data will not be available during the study inclusion period. Following publication, de-identified individual participant data underlying the published results, together with the study protocol and statistical analysis plan, will be made available on reasonable request to the corresponding author.

## Supplementary material

10.1136/bmjopen-2026-120820 online supplemental file 1 Distribution of mPAP at baseline (**T0**) and study endpoint (**T1**) with applied NI margin. Mean mPAP shifts from 23.6 mmHg (T0, green) to 27.6 mmHg (T1, red), with the NI margin of 4 mmHg. The 30 mmHg line, marking the threshold for PH medication initiation, and the 38 mmHg line, marking the threshold associated with worse long-term survival, are shown with black vertical lines. The 97.5th percentile of individual variation (**T1**) is 37.4 mmHg (mean+2σ). mPAP, mean pulmonary artery pressure; NI, non-inferiority; PH, pulmonary hypertension; μ, mean; σ, SD deviation.
