# Supplementary material for: STOP-RIO study: protocol for a randomised, controlled, open-label non-inferiority trial to test the safety of discontinuation of riociguat after balloon pulmonary angioplasty in chronic thromboembolic pulmonary hypertension
Source: BMJ Open. 2026 Aug 7;16(8):e120820. doi: 10.1136/bmjopen-2026-120820 (PMC13475337; doi:10.1136/bmjopen-2026-120820)
Supplement: Supplementary file 1 [file bmjopen-2026-120820.reviewer_comments.pdf]

PEER REVIEW HISTORY

BMJ Open publishes all reviews undertaken for accepted manuscripts. Reviewers are asked to complete a checklist review form and are provided with free text boxes to elaborate on their assessment. These free text comments are reproduced below.

ARTICLE DETAILS

Title (Provisional)

Protocol of the STOP-RIO study - A randomized, controlled, open-label Non-inferiority Trial to Test the Safety of Discontinuation of Riociguat after Balloon Pulmonary Angioplasty in Chronic Thrombo-Embolic Pulmonary Hypertension

Authors

Rodenburg, Tamara Carina; Boerman, Sanne; Post, Marco; Bogaard, Harm-Jan; Aman, Jurjan

---

VERSION 1 - REVIEW

**Reviewer**	**1**
**Name**	**Coghlan, John**
**Affiliation**	**Royal Free London NHS Foundation Trust, Cardiology**
**Date**	**04-Jul-2026**
**COI**	**None**

Rodenburg et al have submitted a protocol for publication. They propose an single-blind non inferiority trial of Riociguat withdrawal in patients after BPA.

The included patients will have a mPAP <30mmHg which is generally thought to be a level associated with an excellent long-term prognosis. They calculate based on published data that mPAP is unlikely to exceed 38mmHg which has been associated with less favourable outcomes among post PEA patients – whether this is the appropriate cut point in post BPA patients has not been established.

Data from multiple large-scale registries suggest that in general prognosis is adversely affected once the mPAP exceeds 20mmHg. So there is a degree of uncertainty about the safety of the chosen safety endpoints.

The Therapy-Hybrid BPA study showed not just a better exercise cardiac output, but also superior VO2 max, thus a better exercise capacity in a similar population when Riociguat was introduced post successful BPA. Since Riogiguat reduces mPAP by approximately 11% pre BPA(Open Heart. 2026 Jun 8;13(1):e004169) – the population may have a different starting point to the Japanese cohort with a drug related lower mPAP post BPA.

The partially open-label design may further confound interpretation, but if the findings in respect of mPAP and proBNP are positive this should be less of an issue. If however the trial is negative – interpretation is difficult.

The health economic data is interesting, however, without longer-term follow-up, extrapolation of findings may not be valid.

The governance issues are adequately addressed – Ethics approval, trial registry, funding, and safety are all addressed.

The statistical plan appears robust, though the per-protocol analysis may be a further confounder, patients that require re-initiation of therapy may have had adverse haemodynamic changes that are only partially rescued by re-initiation. Alternately this may mask the adverse effects of therapy cessation, early EOT assessment before restarting could overcome this issue.

The question arises if the trial shows no difference at rest – does this support cessation of therapy? Long-term outcome, and peak effort tolerance will not have bee addressed, so perhaps only suggestive evidence rather that proof.

Conclusion: The trial addresses and unmet need, however will provide rather limited evidence.

**Reviewer**	**2**
**Name**	**Hosokawa, Kazuya**
**Affiliation**	**Kyushu University**
**Date**	**05-Jul-2026**
**COI**	**None**

General Comments:

The authors present a protocol for the STOP-RIO study, a randomized, open-label, non-inferiority trial investigating the safety and efficacy of withdrawing riociguat in patients with CTEPH who have been successfully treated with balloon pulmonary angioplasty (BPA). This is a well-conceived study that addresses a highly important clinical question and an unmet need in the post-BPA management of CTEPH. Given that the study protocol has already been thoroughly reviewed and funded, the intent of my review is not to request fundamental changes to the study design. Rather, my comments are provided constructively to further enhance the transparency of the manuscript and to strongly recommend pre-specifying an additional exploratory analysis that can still be incorporated into the statistical analysis plan to maximize the clinical utility of your findings.

Major Comments:

1. Impact of BPA Completeness and Baseline mPAP on Trial Success The clinical success of riociguat withdrawal will heavily depend on the "completeness" of the preceding BPA procedures. Patients with highly successful revascularization and lower baseline mPAP are theoretically less likely to experience a clinically significant rebound in mPAP upon drug withdrawal compared to those with higher baseline mPAP (mild residual PH) (Ref. 1). To provide actionable clinical insights into which patient phenotypes can safely discontinue riociguat, I strongly recommend adding a pre-specified exploratory subgroup analysis stratified by baseline mPAP (e.g., baseline mPAP < 25 mmHg vs. ≥ 25 mmHg) to the Statistical Analysis Plan. Incorporating this analysis at this stage would greatly enhance the scientific and practical value of the trial. (Ref. 1) Hosokawa K, Yamasaki Y, Abe K. Technical Considerations for Performing Safe and Effective Balloon Pulmonary Angioplasty in Patients with Chronic Thromboembolic Pulmonary Hypertension. Interventional Cardiology Clinics, 2023; 12, 367-380.

2. Rationale for the Hierarchical Testing Order of Secondary Endpoints In the statistical analysis section (and Table 1), the hierarchical order for secondary endpoints is set as NT-proBNP followed by 6MWD. From a clinical perspective, once the hemodynamic safety of withdrawal is confirmed (the primary endpoint, which is based on a threshold for long-term survival), demonstrating that withdrawal does not worsen a patient's functional capacity (assessed by 6MWD or CPET) is arguably the most meaningful patient-centric outcome. Placing a surrogate biomarker (NT-proBNP) ahead of 6MWD in a fixed-sequence testing procedure carries the risk that if non-inferiority is not met for NT-proBNP, the results for 6MWD cannot be formally claimed as statistically significant. Please provide a clear clinical or statistical rationale in the manuscript for prioritizing NT-proBNP over 6MWD in this sequence.

3. Handling of Missing Data and Lack of an Independent Endpoint Adjudication Committee The protocol states that "missing data will not be imputed." In an open-label non-inferiority trial, using a complete-case analysis can introduce a severe anti-conservative bias. For instance, if patients in the withdrawal group experience clinical worsening of CTEPH and consequently drop out before the 16-week RHC, simply excluding these missing data makes it easier to inappropriately claim non-inferiority. To ensure robustness, please consider pre-specifying a conservative imputation strategy (e.g., worst-case imputation) for dropouts explicitly caused by clinical worsening. Furthermore, since this is an open-label trial without an independent endpoint adjudication committee to blindly assess clinical worsening, I recommend explicitly mentioning this absence of a blinded adjudication committee as a potential source of bias in the "Strengths and limitations of this study" section to enhance protocol transparency.

4. Clinical Justification of the Non-Inferiority Margin Threshold

The authors justify the 4 mmHg non-inferiority margin by arguing that the predicted maximum mPAP (37.4 mmHg) remains below the 38 mmHg threshold for poor long-term

prognosis. However, it is conventionally and widely recognized (e.g., Riedel M et al., Chest 1982) that an mPAP > 30 mmHg is a standard indicator of poor long-term prognosis in pulmonary hypertension. Permitting the mPAP to potentially rebound beyond 30 mmHg (up to 37.4 mmHg) under the assumption of safety may be overly optimistic. Please address this discrepancy in the Discussion section. A stronger clinical rationale for using 38 mmHg rather than the conventional 30 mmHg as the safety threshold should be provided, or this optimistic assumption regarding the non-inferiority margin should be acknowledged as a limitation.

Minor Comments:

1. Simplification of Figure 3 In Figure 3, the timeline for incident patients includes "12mo FU" and "18mo FU," whereas the prevalent patients' timeline does not. While this likely reflects the local routine clinical follow-up schedule at Amsterdam UMC, it is not directly relevant to the trial's specific interventions and may confuse international readers. Please consider simplifying Figure 3 by removing the routine clinical follow-up timings (12mo FU, 18mo FU) and focusing purely on the study-specific timeline (baseline, 8 weeks, and 16 weeks) to improve clarity.

VERSION 1 - AUTHOR RESPONSE

REVIEWER 1

Comments to the Author
Dr. John Coghlan, Royal Free London NHS Foundation Trust
Rodenburg et al have submitted a protocol for publication. They propose an single-blind non inferiority trial of Riociguat withdrawal in patients after BPA.

Comments
C1. "The included patients will have a mPAP <30mmHg which is generally thought to be a level associated with an excellent long-term prognosis. They calculate based on published data that mPAP is unlikely to exceed 38mmHg which has been associated with less favourable outcomes among post PEA patients – whether this is the appropriate cut point in post BPA patients has not been established. Data from multiple large-scale registries suggest that in general prognosis is adversely affected once the mPAP exceeds 20mmHg. So there is a degree of uncertainty about the safety of the chosen safety endpoints."

R1: We thank the reviewer for evaluating the manuscript and for this comment. We agree that no established safety threshold exists for mPAP after BPA and that the prognostic significance of specific mPAP values in this population remains uncertain. The safety boundary of 38 mmHg was based on the available evidence from Cannon et al., although these data were not derived from a post-BPA population.
We added this argument to the Discussion section: *"The non-inferiority margin was primarily chosen to target an upper limit of the 95% confidence interval of the mean mPAP below 30 mmHg after riociguat discontinuation, consistent with current treatment targets after BPA. The 38 mmHg threshold was used to contextualise the expected interindividual variability, based on the best*

available prognostic evidence from patients after PEA. Although this threshold has not been validated in patients after BPA, it currently represents the most appropriate prognostic reference available."

We agree that lower mPAP values are desirable. However, the large-scale registries suggesting that prognosis is adversely affected at mPAP 20 mmHg does not include CTEPH patients, and achieving mPAP as low as <20 mmHg is often not feasible in practice. In addition, there is no evidence (even for the larger PH group) that targeting and mPAP <20mmHg yields prognostic benefit. Whether CTEPH patients with mPAP between 20 and 30 mmHg after vascular intervention require lifelong riociguat therapy remains unknown in this population.

The aim of this study is not to define the optimal mPAP target, but to determine whether riociguat can be safely discontinued in patients with mPAP <30 mmHg after BPA. Importantly, the primary endpoint is the between-group difference in mPAP using a non-inferiority margin of 4 mmHg, rather than the proportion of patients exceeding a safety threshold such as 38 mmHg. Whether discontinuation results in clinically meaningful worsening remains unknown and represents the evidence gap addressed by this study. We will perform prespecified subgroup analyses, including patients with mPAP <25 mmHg, to explore whether the effects differ across haemodynamic risk profiles.
Therefore we added this to the Discussion section*: "To further evaluate the clinical relevance of haemodynamic thresholds after BPA, we aim for an exploratory subgroup analysis to compare patients with an mPAP <25 mmHg and ≥25 mmHg after BPA."*

C2. The Therapy-Hybrid BPA study showed not just a better exercise cardiac output, but also superior VO2 max, thus a better exercise capacity in a similar population when Riociguat was introduced post successful BPA. Since Riociguat reduces mPAP by approximately 11% pre BPA(Open Heart. 2026 Jun 8;13(1):e004169) – the population may have a different starting point to the Japanese cohort with a drug related lower mPAP post BPA.

R2: We thank the reviewer for highlighting the relevant literature. However, we do not fully agree with the interpretation regarding exercise capacity in the Therapy-Hybrid BPA Trial. Although the riociguat continuation group showed a numerically greater improvement in peak VO_2, the between-group difference was not statistically significant (0.72 mL/min/kg, 95% CI −0.63 to 2.07; p=0.29), and the confidence interval of the change of VO2max from baseline to week 16 of both groups included no effect. Also, no significant intergroup differences in 6MWD were found from baseline to week 16. Nevertheless, we agree that exercise capacity is an important clinical outcome and therefore included CPET in our study.

We also agree that differences between Japanese and Western BPA populations may influence the applicability of the Therapy-Hybrid BPA results to a Western population. Patients treated in experienced Japanese centres generally achieve lower post-BPA mPAP values than those reported in Western cohorts, which may affect the impact of riociguat discontinuation. Although the reported haemodynamic response to riociguat appears broadly comparable across studies (approximately 6 mmHg in the Japanese MR BPA study by Kawakami et al. (Lancet Respir Med, 2022) versus approximately 5 mmHg in the French RACE study and the UK CURATE registry), differences in patient characteristics, treatment strategies, and post-BPA haemodynamics limit direct comparison. We clarified this in the Discussion section: *"Differences in patient characteristics, treatment strategies, and haemodynamic responses to BPA may limit the generalisability of these finding to Western populations."*

C3. The partially open-label design may further confound interpretation, but if the findings in respect of mPAP and proBNP are positive this should be less of an issue. If however the trial is negative – interpretation is difficult.

R3. We agree that the open-label design is a limitation, particularly for subjective secondary outcomes. However, because the primary endpoint is an objective haemodynamic measurement, we do not expect the interpretation of either a positive or a negative result to be substantially affected by the open-label design.

C4. The health economic data is interesting, however, without longer-term follow-up, extrapolation of findings may not be valid. The governance issues are adequately addressed – Ethics approval, trial registry, funding, and safety are all addressed.

R4. We thank the reviewer for this comment and agree that a 16-week follow-up is relatively short for a comprehensive health economic evaluation. Therefore, the economic analysis should be considered exploratory and will primarily provide insight into the short-term costs associated with the riociguat discontinuation strategy, including potential cost savings from treatment discontinuation and costs related to treatment re-initiation when required. Longterm costs and cost-effectiveness cannot be reliably assessed within the timeframe of the current study and will require longer follow-up. We therefore intend to perform a long-term follow-up study, including an extended health economic evaluation, to assess the sustainability of both the clinical and economic effects of riociguat discontinuation over time.
We added this to the Limitation section of the Discussion: *"Finally, the 16-week follow-up limits assessment of the long-term clinical and health economic consequences of riociguat discontinuation. Therefore, we aim for a long-term follow-up study, including an extended health economic evaluation."*

C5. The statistical plan appears robust, though the per-protocol analysis may be a further confounder, patients that require re-initiation of therapy may have had adverse haemodynamic changes that are only partially rescued by re-initiation. Alternately this may mask the adverse effects of therapy cessation, early EOT assessment before restarting could overcome this issue.

R5: We thank the reviewer for this important comment and agree that re-initiation of riociguat could complicate the interpretation of the haemodynamic effect of treatment discontinuation. This is why multiple complementary analyses will be performed.

The primary analysis will be performed in the per-protocol population which is the state of the art in non-inferiority trials as it is the most conservative approach. Patients who restart riociguat because of clinical worsening will be considered treatment crossovers and analysed with the continuation group. The main reason for this is that when patients cross over from stopping group to the continuation group (because of clinical worsening) will be analysed according to intention-to-treat, these patient will be considered as stopping (following their randomization) but in fact have experienced the biological effect of riociguat. In that case, the difference in the primary outcome will be underestimated (due to the biological effect of restarting riociguat), and the intention-to-treat approach will be anti-conservative. As a secondary analysis, the primary endpoint will be analysed according to the intention-to-treat principle, in which all patients are analysed according to their original treatment allocation, irrespective of treatment re-initiation. This analysis may mask the therapy effects but reflects the stopping strategy and therefore captures the consequences of attempting riociguat discontinuation.

We planned prespecified exploratory analyses, including a subgroup analysis of subjects who restarted riociguat, compared with the control group. This will assess the impact of re-initiation, particularly whether mPAP values return to baseline within 2 months or are worsened after restarting. We agree that an earlier assessment before treatment re-initiation would be informative. Therefore, patients requiring re-initiation will undergo an additional transthoracic echocardiogram before restarting therapy to document changes in cardiac function and estimated pulmonary haemodynamics. However, we do not consider an additional RHC at that time to be proportionate, given the associated procedural burden, and as treatment should be restarted promptly once predefined criteria for clinical worsening are met.

C6. The question arises if the trial shows no difference at rest – does this support cessation of therapy? Long-term outcome, and peak effort tolerance will not have been addressed, so perhaps only suggestive evidence rather that proof. Conclusion: The trial addresses and unmet need, however will provide rather limited evidence.

R6: We agree that the present study is not designed as a long-term study on the effects of riociguat discontinuation. Rather, it aims to provide evidence regarding the short-term haemodynamic effects of a discontinuation strategy in patients after successful BPA. Exercise capacity will be assessed using the 6MWD and CPET. Although this is not the primary outcome, as specified before, the study is powered to detect possible differences in 6MWD as hierarchised secondary endpoint. In addition, secondary exploratory endpoints include CPET parameters. For this reason the study will provide proof and clinically relevant evidence on effort tolerance. . We are therefore planning a long-term follow-up study to assess the durability of the haemodynamic, clinical and economic effects of riociguat discontinuation.

REVIEWER 2

Comments to the Author
Kazuya Hosokawa, Kyushu University
The authors present a protocol for the STOP-RIO study, a randomized, open-label, non-inferiority trial investigating the safety and efficacy of withdrawing riociguat in patients with CTEPH who have been successfully treated with balloon pulmonary angioplasty (BPA). This is a well-conceived study that addresses a highly important clinical question and an unmet need in the post-BPA management of CTEPH. Given that the study protocol has already been thoroughly reviewed and funded, the intent of my review is not to request fundamental changes to the study design. Rather, my comments are provided constructively to further enhance the transparency of the manuscript and to strongly recommend pre-specifying an additional exploratory analysis that can still be incorporated into the statistical analysis plan to maximize the clinical utility of your findings.

We thank the reviewer for the constructive evaluation of our study protocol. We addressed all comments below and incorporated the recommended additional exploratory analysis into the statistical analysis plan.

Major Comments
C1. Impact of BPA Completeness and Baseline mPAP on Trial Success The clinical success of riociguat withdrawal will heavily depend on the "completeness" of the preceding BPA procedures. Patients with highly successful revascularization and lower baseline mPAP are theoretically less likely to experience a clinically significant rebound in mPAP upon drug withdrawal compared to those with higher baseline mPAP (mild residual PH) (Ref. 1). To provide actionable clinical insights

into which patient phenotypes can safely discontinue riociguat, I strongly recommend adding a pre-specified exploratory subgroup analysis stratified by baseline mPAP (e.g., baseline mPAP < 25 mmHg vs. ≥ 25 mmHg) to the Statistical Analysis Plan. Incorporating this analysis at this stage would greatly enhance the scientific and practical value of the trial.
(Ref. 1) Hosokawa K, Yamasaki Y, Abe K. Technical Considerations for Performing Safe and Effective Balloon Pulmonary Angioplasty in Patients with Chronic Thromboembolic Pulmonary Hypertension. Interventional Cardiology Clinics, 2023; 12, 367-380.

R1: We thank the reviewer for this suggestion and agree that the degree of residual pulmonary hypertension after BPA might influence the effects of riociguat discontinuation. Therefore, we prespecified an exploratory subgroup analysis comparing patients with mPAP <25 mmHg and ≥25 mmHg after BPA. We added this to the Discussion: *"To further evaluate the clinical relevance of haemodynamic thresholds after BPA, we aim for an exploratory subgroup analysis to compare patients with an mPAP <25 mmHg and ≥25 mmHg after BPA."*

C2. Rationale for the Hierarchical Testing Order of Secondary Endpoints In the statistical analysis section (and Table 1), the hierarchical order for secondary endpoints is set as NT-proBNP followed by 6MWD. From a clinical perspective, once the hemodynamic safety of withdrawal is confirmed (the primary endpoint, which is based on a threshold for long-term survival), demonstrating that withdrawal does not worsen a patient's functional capacity (assessed by 6MWD or CPET) is arguably the most meaningful patient-centric outcome. Placing a surrogate biomarker (NT-proBNP) ahead of 6MWD in a fixed-sequence testing procedure carries the risk that if non-inferiority is not met for NT-proBNP, the results for 6MWD cannot be formally claimed as statistically significant. Please provide a clear clinical or statistical rationale in the manuscript for prioritizing NT-proBNP over 6MWD in this sequence.

R2: We thank the reviewer for this thoughtful comment. We agree that functional capacity is an important outcome. The hierarchical testing order was chosen because NT-proBNP is an objective biomarker that more directly reflects the haemodynamic status, thereby complementing the primary haemodynamic endpoint (mPAP). In contrast, 6MWD might be influenced by comorbidities or the open-label design of the study. Furthermore, although both NT-proBNP and 6MWD were incorporated into the sample size calculation, the planned sample size of 74 participants provides greater statistical power to detect differences in NT-proBNP than in 6MWD. Together with its more objective nature, this formed the rationale for prioritising NT-proBNP in the hierarchical testing.
We added this to the Sample Size Calculation in the Methods section; *"NT-proBNP was prioritised in the hierarchical testing strategy because it is an objective biomarker that reflects haemodynamic status. Also, the planned sample size provides greater statistical power for NT-proBNP than for 6MWD."*

C3. Handling of Missing Data and Lack of an Independent Endpoint Adjudication Committee The protocol states that "missing data will not be imputed." In an open-label non-inferiority trial, using a complete-case analysis can introduce a severe anti-conservative bias. For instance, if patients in the withdrawal group experience clinical worsening of CTEPH and consequently drop out before the 16-week RHC, simply excluding these missing data makes it easier to inappropriately claim non-inferiority. To ensure robustness, please consider pre-specifying a conservative imputation strategy (e.g., worst-case imputation) for dropouts explicitly caused by clinical worsening. Furthermore, since this is an open-label trial without an independent endpoint adjudication committee to blindly assess clinical worsening, I recommend explicitly mentioning this absence of a blinded adjudication committee as a potential source of bias in the "Strengths and limitations of this study" section to enhance protocol transparency.

R3: We thank the reviewer for these important comments. As patients who restart riociguat due to clinical worsening remain in follow-up, we expect limited missing data. Nevertheless, to address this concern, we have added a conservative approach whereby patients who withdraw from follow-up because of clinical worsening before formal clinical worsening assessment at week 8, or for whom the reason for withdrawal is unknown, will be classified as having clinical worsening. Furthermore, the number of patients requiring riociguat re-initiation due to clinical worsening will be reported as a secondary endpoint, providing full transparency regarding treatment failure.
We added this to the Analysis set section of the Methods: *"However, participants who withdraw before the week 8 clinical worsening assessment because of clinical deterioration, or for whom the reason for withdrawal is unknown, will be conservatively classified as having experienced clinical worsening."*

We agree that the absence of an independent blinded endpoint adjudication committee is a limitation. However, riociguat re-initiation due to clinical worsening is based on predefined protocol criteria, and patient safety is further safeguarded by a prespecified interim analysis with adaptive randomisation and predefined criteria for early study termination if the rate of clinical worsening exceeds the predefined safety threshold. Therefore we believe that an independent adjudication committee would have limited additional value. A rationale for this was added to the Limitation section of the Discussion: *"In addition, no independent blinded endpoint adjudication committee was included; however, clinical worsening and riociguat re-initiation are based on predefined protocol criteria."*

C4. Clinical Justification of the Non-Inferiority Margin Threshold
The authors justify the 4 mmHg non-inferiority margin by arguing that the predicted maximum mPAP (37.4 mmHg) remains below the 38 mmHg threshold for poor long-term prognosis. However, it is conventionally and widely recognized (e.g., Riedel M et al., Chest 1982) that an mPAP > 30 mmHg is a standard indicator of poor long-term prognosis in pulmonary hypertension. Permitting the mPAP to potentially rebound beyond 30 mmHg (up to 37.4 mmHg) under the assumption of safety may be overly optimistic. Please address this discrepancy in the Discussion section. A stronger clinical rationale for using 38 mmHg rather than the conventional 30 mmHg as the safety threshold should be provided, or this optimistic assumption regarding the non-inferiority margin should be acknowledged as a limitation.

R4: We thank the reviewer for this important comment. We agree that the prognostic significance of mPAP values between 30 and 38 mmHg after BPA remains uncertain. Importantly, the 4 mmHg non-inferiority margin was primarily chosen to ensure that the upper limit of the 95% confidence interval of the mean mPAP remains below 30 mmHg, consistent with current treatment targets after BPA. The 38 mmHg threshold was used to contextualise the expected interindividual variability, based on the best available prognostic evidence from patients after PEA. We acknowledge that this threshold has not been validated in patients after BPA and revised the Discussion to clarify this rationale.

"The non-inferiority margin was primarily chosen to target an upper limit of the 95% confidence interval of the mean mPAP below 30 mmHg after riociguat discontinuation, consistent with current treatment targets after BPA. The 38 mmHg threshold was used to contextualise the expected interindividual variability, based on the best available prognostic evidence from patients after PEA. Although this threshold has not been validated in patients after BPA, it currently represents the most appropriate prognostic reference available."

Minor Comments:
C1. Simplification of Figure 3 In Figure 3, the timeline for incident patients includes "12mo FU" and "18mo FU," whereas the prevalent patients' timeline does not. While this likely reflects the local

routine clinical follow-up schedule at Amsterdam UMC, it is not directly relevant to the trial's specific interventions and may confuse international readers. Please consider simplifying Figure 3 by removing the routine clinical follow-up timings (12mo FU, 18mo FU) and focusing purely on the study-specific timeline (baseline, 8 weeks, and 16 weeks) to improve clarity.

C1: We thank the reviewer for this suggestion and agree that the routine clinical follow-up schedule is specific to our local practice and is not essential for understanding the study design. To improve clarity and avoid potential confusion, we removed Figure 3 from the manuscript.
